# *PLAG1* rearrangement may be an oncogenic driver in a subset of sporadic cardiac myxomas: a case–control study

**DOI:** 10.3389/fcvm.2026.1745004

**Published:** 2026-05-20

**Authors:** Fausto Maffini, Daniela Lepanto, Eleonora Pisa, Eisa De Camilli, Valentina Catto, Chiara Zanetti, Simona Pessina, Corrado Carbucicchio, Fabio Pagni, Nicola Fusco

**Affiliations:** 1 Laboratory of Pathology and Somatic Molecular Diagnostics, European Institute of Oncology IRCCS, Milan, Italy; 2 Department of Clinical Electrophysiology and Cardiac Pacing, Centro Cardiologico Monzino, IRCCS, Milan, Italy; 3 Department of Pathology, School of Medicine, S. Gerardo Hospital, IRCCS, Italy and State University of Milan Bicocca, Monza/Milan, Italy; 4 Department of Oncology and Hemato-Oncology, School of Medicine, State University of Milan, Milan, Italy

**Keywords:** cardiac myxoma, Carney complex, heart tumor, MYC amplification, PLAG1 rearrangement, silver Russel syndrome, sporadic myxoma

## Abstract

**Background:**

Sporadic cardiac myxoma (SCM) is the most common primary tumor of the heart; however, its molecular pathogenesis remains poorly understood. Unlike the familial forms associated with the Carney complex (CNC), SCM lacks well-defined genetic alterations. Recognizing the established role of pleomorphic adenoma gene 1 (PLAG1) rearrangements in developmental syndromes with cardiac anomalies and in neoplasms characterized by prominent myxoid stroma, we investigated whether similar genetic events occur in SCM. In parallel, we evaluated MYC amplification, considering its known association with the malignant transformation in the mesenchymal tumors.

**Methods:**

We retrospectively analyzed 14 SCM cases using fluorescence *in situ* hybridization (FISH) to assess PLAG1 rearrangement and MYC amplification. Histological and immunohistochemical analyses were performed. Fifteen normal salivary gland tissues were used as controls for the FISH-based cutoff definition. The statistical tests included the ROC curve with AUC and Youden index, t-test, Welch's test, *F*-test, along with corrections using the Bonferroni and Benjamini–Hochberg methods.

**Results:**

Classical PLAG1 break-apart signals were detected in 35.7% (5/14) of the SCMs. Atypical isolated 5′ (red) and 3′ (green) signals were frequently observed (64.3% and 85.7%, respectively), potentially reflecting unbalanced translocations or copy number variation. No MYC amplification was observed in any of the cases. Statistical analysis positively supported a >0.5% threshold for PLAG1 Break-Apart (PLAG1-BA). The histological features were consistent with typical SCM morphology, and there was no evidence of malignancy.

**Conclusion:**

This study reveals PLAG1 rearrangement in a subset of SCMs, suggesting potential molecular similarities with other tumors characterized by an abundant myxoid stroma. The absence of MYC amplification reinforces the benign nature of these neoplasms. These findings raise new hypotheses regarding the pathogenesis of SCM and warrant further investigation into cardiac myxoid tumors.

## Introduction

Sporadic cardiac myxoma (SCM) is the most common tumor of the heart, typically originating in the atrial chambers, most frequently in the left atrium. Although the pathogenesis of the Carney complex (CNC) is well understood (specifically, through the mutations in the protein kinase cAMP-dependent type I regulatory subunit alpha (PRKAR1A) located at 17q22-24 in CNC type 1, or through involvement of chromosome 2p16 in CNC type 2), these alterations are absent in sporadic forms (1–7). Patients with CNC exhibit cutaneous and endocrine manifestations, in addition to visceral manifestations, with an increased probability of developing cardiac myxoma in 20%–40% of cases (1–5). In contrast, SCM is extremely rare, accounting for 0.5–1 per million people every year, and its molecular drivers remain poorly understood. Despite its rarity, SCM remains the most frequently encountered cardiac tumor, and its etiology outside familial syndromes is largely unknown (8–10).

The involvement of pleomorphic adenoma gene 1 (PLAG1) rearrangements (Chr 8q12) is well documented in syndromes associated with cardiac and developmental anomalies, such as CNC and Silver–Russell syndrome (SRS) (11, 12). In addition, these alterations are frequently observed in tumors with a prominent myxoid component [e.g., pleomorphic adenoma/carcinoma ex-pleomorphic adenoma (PA/Ca-Ex-PA) of the salivary gland, myxoid lipoblastoma, and matrix-producing breast metaplastic carcinoma] (13–15). We hypothesized that PLAG1 rearrangement might also contribute to the pathogenesis of SCM. Therefore, we investigated the presence of PLAG1 rearrangements in SCM using fluorescence *in situ* hybridization (FISH). In addition, we investigated the involvement of MYC (Chr 8q24), considering its known association with malignant progression in mesenchymal tumors, to better characterize the genetic profile of this rare, but important and distinctive cardiac neoplasm.

## Materials and methods

### Morphological and immunohistochemical analyses

We identified 15 SCM cases between 2020 and 2022. Patients were recruited following consent in accordance with the Helsinki Declaration guidelines. Written informed consent was obtained from each patient for the study, and approval was granted by the ethics committee (IEO No. ID-5384; IEO code: L2-596). FFPE blocks were collected from the anatomic pathology department archive at the European Institute of Oncology (IEO, Milan, Italy) and clinical data from Centro Cardiologico Fondazione Monzino (CCFM, Milan, Italy). For diagnostic purposes, H&E staining, immunohistochemical staining, and fluorescent *in situ* hybridization (FISH) were performed. Subsequently, the FFPE blocks were cut into 5 µm-thick sections. After the sections were deparaffinized and dehydrated, the slides were stained with standard H&E stain, dehydrated in alcohol, and cover-slipped. Immunohistochemical staining for keratin AE1–AE3 (Novocastra, clone AE1:AE3, dilution 1:50), CD34 (Novocastra, clone END/10, dilution 1:400), and Calretinin (SWANT, polyclonal, dilution 1:1,000) was used following the factory guidelines for a brown end product that stains the cytoplasm for keratin and for CD34, and the nucleocytoplasm for calretinin. Internal and external controls were used to avoid false-positive and negative staining.

### FISH analysis

FISH was performed using a dual-color break-apart (BA) PLAG1 probe (Empire Genomics, Buffalo, NY, USA). The PLAG1 probe is a dual-color break-apart probe consisting of a 265-kb 3′-centromeric side labeled green and a 289-kb 5′-telomeric side labeled orange. A 4-μm-thick section of FFPE was air-dried and oven-baked for 30 min at 60°C. FISH was performed in accordance with the manufacturer's technical instructions; 10 μL of PLAG1 mix probe was added, and the slides were cover-slipped and sealed. The slides were denatured for 3 min at 83°C and then hybridized overnight at 37°C on a Thermo-Brite. Following hybridization, coverslips were removed by placing the slides in a room temperature bath of 2×SSC. After hybridization, the slides were transferred to a solution heated to 0.4×SSC with 0.3% IGEPAL at 72°C for 2 min, and then rinsed in 2×SSC with 0.1% IGEPAL at room temperature. A two-fusion signal pattern indicates no rearrangement involving the PLAG1 gene, whereas a distant separation between the orange and green signals indicates PLAG1 involvement. For MYC amplification, the threshold limit of the MYC/CEP8 ratio was set at ≥2. Orange staining was used to evaluate the MYC locus at 8q24, and green staining was used to evaluate the centromeric 8 chromosome (8-CEP) (16).

### Statistical analysis

To prevent laboratory errors, we evaluated 15 cases of normal salivary tissue as control cases (CC) for PLAG1-BA, following the guidelines of the European Journal of Human Genetics (17). We performed a descriptive statistical analysis (Supplementary Data) followed by an ROC curve with an AUC and Youden's index to obtain a threshold value. After obtaining Youden's index, we evaluated the Student’s *t*-test, a modified Welch's *t*-test, and the *F*-test (Fisher–Snedecor). We corrected the results using the Bonferroni and Benjamini–Hochberg tests to reduce the rate of false positives (see Supplementary Data). Finally, we evaluated the mean and variance differences between the two groups.

## Results

### Clinicopathological features

**Figure 1:**
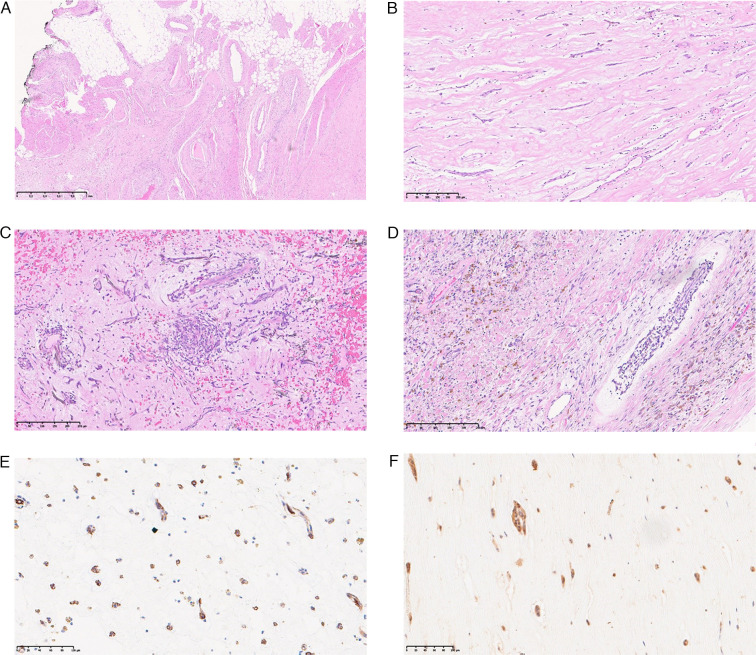
(**A**–**F)** Histological features of cardiac myxoma. (**A**–**C**) H&E staining is characterized by a myxoid background with diffuse hemorrhagic foci and an aggregate of neoplastic spindle-stellate cells. There is involvement of the muscular wall of all of the atrial muscle layers up to the adipose pericardial tissue in **A**; scale bar, 1 mm. (**B**–**D**) Aggregate of neoplastic cells without overt atypia in a myxoid background; scale bar, 250 µm. (**D**) Hemosiderin pigment is present, as well as involvement of the perivascular connective tissue; scale bar, 250 µm. (**E**) Neoplastic cells staining for CD34; scale bar, 100 µm, whereas the calretinin staining of the neoplastic cells is shown in **F**; scale bar, 100 µm. All images show a scale bar in the bottom left corner.

In the vast majority of cases, the tumor was located in the left atrium, with only one case arising in the mitral valve (Table 1: case no.1). The tumor was grossly characterized by a translucent brown mass with a “jelly-like appearance,” connected to the heart wall by a peduncle or broad base, involving the heart muscles, and in a few cases, the pericardial fatty tissue (Figure 1A). The average tumor diameter was 3.1 cm (range: 1.2–4.3 cm) (Table 1). Additionally, one case showed a synchronous lymphoma (Table 1: case no.1) and was removed from the biological analysis. We found more affected females than males, with a ratio of F:M 2.75:1 and 11F and 4M. The average age was 65.9 years (range: 43–87 years) (Table 1). The prominent tissue features were a myxoid background with thin vessels, and areas rich in cells and others devoid of them (Figures 1A–F). The cells of the myxoma were thin and fusate stellate with dark, ovoid, or circular nuclei exhibiting fine chromatin, and the nucleolus was barely observable (Figures 1A–F). Occasionally, hemorrhagic foci and Gamna Gandy bodies were observed within the framework. Very rarely, some tumors are associated with other neoplasms such as lymphoma (Table 1: case no. 1) (18). No mitotic figures, necrosis, or cytological atypia suggestive of malignancy were observed in the study group (Figures 1A–F).

**Table 1:** Statistics for the 14 patients with myxoma.

ID case	Sex	Age (Y)	Odd	Place	Max-diameter (cm)
1	F	45	Lymphoma	MV	3.5
2	F	70		A	4
3	M	71		A	1.7
4	F	54		LA	4
5	F	54		LA	4
6	M	72		LA	4.3
7	F	82		A	3
8	F	78		A	1.2
9	M	87		n.r.	4
13	F	53		A	1.8
15	F	43		LA	3
16	M	72		A	3
17	F	74		LA	1.9
18	F	76		A	3.5
19	F	58		LA	2.2
Average	F (73.3%)	65.9			3.125

One case showed a combined lymphoma “ID-1.” LA, left atrium; MV, mitral valve; N.R., not reported; A, atrium.

**Table 2:** Genes observed and reported altered in 14 cases of SCM with relative (%)frequency after a correction with a threshold value >0.5% for PLAG1-BA.

ID case	Gene	% SplitT	% Single (5’)	% Single (3’)	GCN	Ratio
1	*MYC* AMP				ne	ne
	*PLAG1*	ne	ne	ne		
2	*MYC* AMP				1.9	1
	*PLAG1*	0	2	4		
3	*MYC* AMP				2	1
	*PLAG1*	1.5	2	5		
4	*MYC* AMP				2.1	1.1
	*PLAG1*	0	0	2		
5	*MYC* AMP				2.4	1.1
	*PLAG1*	0	2	0		
6	*MYC* AMP				2.1	1
	*PLAG1*	0.59	4	1		
7	*MYC* AMP				2.6	1
	*PLAG1*	0	0	4		
8	*MYC* AMP				2.1	1
	*PLAG1*	0	0	2		
9	*MYC* AMP					1
	*PLAG1*	0	0	0		
13	YC AMP				2	1.1
	*PLAG1*	1.5	2	3		
15	*MYC* AMP				1.8	1.2
	*PLAG1*	0.62	0	3		
16	*MYC* AMP				1.7	0.9
	*PLAG1*	0	2	1		
17	*MYC* AMP				1.9	1
	*PLAG1*	0	2	2		
18	*MYC* AMP				2.2	1
	*PLAG1*	2.76	1	8		
19	*MYC* AMP				1.9	1
	*PLAG1*	0	2	1		

Here, there are other “atypical” alterations observed as gene copy gain for red and green signals involving both genes. It never showed MYC amplification. Legend: NE, not evaluable; SPLIT, percentage of signal break-apart of the gene: percentage of genes with a specific break; a yellow marker indicates a normal gene, while the presence of both red and green markers indicates a pathological gene with a translocation (break or split); MYC AMP, MYC amplification; GCN, gene copy number. One case was eliminated from the analysis because it was combined with lymphoma, and the biological data were not evaluable.

### PLAG1 alteration significance and diagnostic threshold

After the ROC curve, an AUC of 0.63 was reported. This result shows that the PLAG1 test is not powerful enough to differentiate between the two populations, most likely due to the small number of cases. However, a Youden's index found that a 0.5% rearrangement could be an acceptable cutoff (sensibility of 0.642 and a specificity of 0.67), suggesting that a value >0.5% could be used as a threshold (Supplementary Data).

After calculating a threshold value from 15 salivary gland CC, only two showed PLAG1-BA signals (0.8% and 1% of cells), while the remainder were considered negative (value ≤0.5% of threshold). Five SCM cases were rearranged for PLAG1 (range:0.59%–2.76%) (Table  2) (19–21). Subsequent statistical analyses revealed that neither the Student's *t*-test nor the modified Welch's *t*-test reached statistical significance (*p* = 0.059 and *p* = 0.068, one-tailed, respectively) (Table 3). Furthermore, only the *F*-test showed a significant difference in variance between the SCM and CC (*p* = 0.0004). Correction with Bonferroni and Benjamini–Hochberg methods, with the aim of avoiding a false-positive rate, was performed (corrected *p* = 0.017 and *p* = 0.017, respectively). These results support the variability of our *F*-test results, which suggests a significant difference between the two sample groups and that the variance found in PLAG1-BA is not due to chance or a technical error (19–21).

**Table 3:** Descriptive statistical differences between the 14 SCM cases and the 15 control cases (CC).

Descriptive analyses of SCM and CC		
	SCM	CC
n.	14	15
-*Σ*x	6.97	1.8
-*Σ*x^2^	12.8501	1.640
SS	9.38	1.424
Mean	0.4979	0.12
*s*	0.84	0.31
*S* ^2^	0.72	0.11

Standard deviation (*s*); SS, square sum; variance (*S*^2^); *Σ*x, sum of value; *Σ*x^2^, sum of the square value.

### PLAG1 rearrangement and a lack of MYC amplification

**Figure 2:**
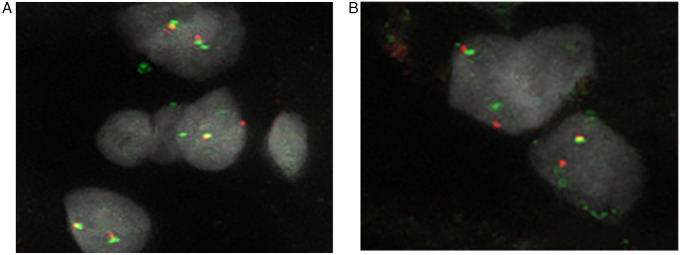
(**A**, **B**) Rearrangements of PLAG1 as signal split and break-apart in two easily evaluable cases. The normal fusion signal is yellow, whereas the split red and green signals are spaced out from each other.

PLAG1 rearrangement was detected by FISH BA analysis in five of 14 evaluable SCM cases (35.7%). In addition to these classical BA patterns, single 5′ red (R) and 3′ green (G) signals were observed in 64.3% and 85.7% of the cases, respectively (Figures 2A,B). These atypical signal patterns may indicate unbalanced translocations or gains of heterozygosity, although their biological significance is uncertain. Importantly, no cases showed PLAG1 gene amplification (Table 4). Simultaneously, FISH analysis for MYC showed no amplification in any tumor (Tables 2, 4), which is consistent with the histologically benign nature of the lesions and contrasts with reports of MYC involvement in more aggressive cardiac neoplasms.

**Table 4:** Features of all-modified genes observed in our 14 cases with a relative frequency.

Genes evaluated among 14 cases	No. of cases	Frequency observed (%)
*PLAG1* BA	5	35.7
*MYC* amp	0	0
Single 5’ *PLAG1*	9	64.3
Single 3’*PLAG1*	12	85.7

Cases with a threshold >0.5% of PLAG1-BA were considered “positive” or rearranged.

## Discussion

Sporadic heart myxoma arises in the heart chambers with more frequency in the left atrium around the “fossa-ovale.” In this location, the incidence of SCM is very high because of the abundance of multipotential cells, which are endothelial-derived cells suspected to be the origin of the tumor (22). Although it is a benign tumor, malignant transformation has rarely been reported in the scientific literature. From a physician's perspective, the origin site is prone to changes in normal cardiac function, leading to sudden death or thromboembolic events, sometimes with dire consequences (23–26). Currently, this tumor is less known due to its rarity and the biological changes underlying its histology, which have sparked debate in the medical world. Histopathology of the tumor is characterized by interspersed bland fusate-stellate cells that typically exhibit strong CD34 and calretinin staining in rich myxoid stroma. Myxoid stroma is the most important histopathological feature. In addition, it mainly shows scant or absent mitosis, diffuse foci of hemorrhage, and sometimes Gamna Gandy bodies due to old organized hemorrhagic foci (Figures 1A–E). Tumoral myxoid matrices are observed in many tumors, including breast matrix-producing carcinoma and many others of mesenchymal origin (13–15). Although the cause of inherited myxoma is well established and linked to the germline mutation of PRKAR1A, as reported in CNC 1 and 2, sporadic cases lack underlying genetic alterations. Patients with this syndrome have an increased probability of developing cardiac myxoma due to heterozygous mutations of the PRKAR1A gene on Chr-17q22-24 (CNC 1) and Chr-2p16 (CNC 2) (3, 5). An inherited autosomal-dominant pattern increases the probability of developing cardiac myxoma without a preferred anatomical heart chamber, especially in young individuals (1, 5). In CNC, the mutation of PRKAR1A upregulates IGF-I mRNA, which has been observed in a significant number of cases of breast, stomach, thyroid, lung, and colorectal cancers (27–33). In contrast, the etiopathogenesis of SCM is poorly understood and remains debated. It has been observed to affect women more than men, particularly at older ages. In rare cases, myxoma components exhibit chondroid metaplasia. Thus, myxoid stroma could be an important histological clue for a comprehensive analysis of the origin. Another peculiarity is the presence of heart malformations in SRS, which shows the involvement of PLAG1–PLAG2. Mutation of these genes followed by downregulation of IGF-I and IGF-II and a reduction in growth (11, 34). PLAG1 is a zinc finger protein that encodes a seven-domain C2H2 protein that suppresses cell growth. PLAG1 is a protein that regulates various functions, such as proliferation and differentiation, during embryogenesis (https://www.ncbi.nlm.nih.gov/gene/5325) (35). The loss of these functions in embryogenesis is responsible for SRS with heart malformations and reduced growth. This syndrome is characterized by dysmorphism, short stature, and a triangular face, and may involve the heart. The most extensively studied tumor with a myxoid component similar to heart myxoma is the pleomorphic adenoma/carcinoma of the salivary gland (PA/Ca-Ex-PA). Both are characterized by a chondromyxoid matrix with different percentages of epithelial and basal cell content. They are biologically characterized by rearrangements of the PLAG1-HMGA2-IGF-II axis in a significant number of cases, which upregulate IGF-II and acquire neoplastic features (36–39). Alterations of PLAG1 were observed and reported in many other tumors with myxoid stroma. For instance, breast metaplastic carcinoma matrix-producing, myoepithelial tumors sometimes exhibit myxoid stroma, as do other tumors (13–15, 40, 41). In our study, PLAG1 was found to be rearranged in five instances as a classical BA, while the extra-green (3’) or extra-red (5’) FISH signal could be due to unbalanced translocation with a gain of heterozygosity or a deletion (Figures 2A,B).

PLAG1 gain in copy number has been observed and reported in PA/Ca-EX-PA and other tumors (13–15, 39, 41). Compared to salivary PA/Ca-EX-PA with SCM, the rearrangement of PLAG1 may affect IGF-II via High Mobility Group AT-Hook 2 (HMGA2). This pathway is the most frequent pathway involved in PA/Ca-Ex-PA (37). It can be inferred that PLAG1 rearrangement through HMGA2 results in the upregulation of IGF-II. This may serve as a pivotal oncogenic protein in the development of this tumor. In this axis, the rearrangement of PLAG1 was followed by neoplastic growth, proliferation, and acquisition of oncogenic properties. Karen Hansen et al. demonstrated that when PLAG1 and its paralog, PLAGL1, were injected into nude mice, they induced a rapid neoplastic growth at the site of injection. From a histopathological perspective, this growth resembled a fibrosarcoma without metastatic dissemination (42). It is noteworthy that in CNC, there is an upregulation of IGF-I, whereas in sporadic myxoma, the action is asserted by the upregulation of IGF-II in 35.7% of our cases. Both IGF-I and IGF-II belong to a family of mitogenic proteins associated with growth, progression, and tumor survival (42).

Although the presence of PLAG1 rearrangement was an unexpected finding, the absence of MYC amplification in our results was expected (Figures 2A,B). MYC amplification has been reported in other cases of heart myxoma in the medical literature, as amplification of MYC can confer a potential malignancy in a tumor (35, 42, 43). PLAG1 rearrangement has also been reported in other tumors, with MYC potentially associated with malignant features, while PLAG1 has not been consistently linked to malignancy (14, 44). The atypical feature of PLAG1, characterized by GCG, involving both the red (5’) and green (3’) signals, is still a subject of debate, and the significance of these Genes Copy Gain (GCG) patterns is still unknown, posing challenges for their interpretation (45).

This study had several limitations that must be acknowledged when interpreting the findings. First, the limited number of SCM cases analyzed reflects the rarity of this neoplasm and inevitably restricts the statistical power of our observations. Although our data suggest a potential role for PLAG1 rearrangement in a subset of cases, the small sample size and lack of complementary molecular analyses (e.g., RNA sequencing and/or comprehensive genome profiling) limit the generalizability and robustness of this conclusion. Similarly, the absence of MYC amplification, while consistent with the benign histology of our cases, cannot be used to exclude its involvement in other SCM subsets or in rare malignant transformations reported in the literature. Finally, the suggestion that PLAG1 rearrangement may activate IGF-II via a mechanism analogous to that in other myxoid tumors is biologically plausible but remains speculative, as we did not assess IGF-II expression or activation directly. Furthermore, the possibility of medical treatment aimed at inhibiting the IGF receptor by using BMS754807 is worth highlighting. This treatment has a significant half-maximal inhibitory concentration (IC50) effect linked to PLAG1 rearrangement, as reported by Zheng et al. (46) and in a phase I clinical trial (NCT00793897) (47).

Functional studies are required to elucidate whether this axis is truly active in the SCM.

## Conclusion

This study raises important and novel questions about SCM pathogenesis. The unexpected detection of PLAG1 rearrangements in SCM opens the door to new research questions and raises the possibility of previously unrecognized molecular subtypes within this rare tumor. Although our findings provide evidence that a subset of SCMs may harbor alterations similar to those observed in other tumors with myxoid stroma, the clinical implications of these alterations remain unclear. The absence of MYC amplification reinforces the benign behavior of the SCM and further differentiates it from tumors with malignant potential. The parallels with genetic syndromes, such as CNC and SRS, offer a conceptual framework for exploring shared molecular pathways, especially those involving IGF signaling. However, these associations are currently speculative. Future multicenter studies with larger cohorts and comprehensive molecular profiling are crucial to validate our findings.

## Data Availability

The original contributions presented in this study are included in the article/Supplementary Material, further inquiries can be directed to the corresponding author.
