# Supplementary material for: PLAG1 rearrangement may be an oncogenic driver in a subset of sporadic cardiac myxomas: a case–control study
Source: Front Cardiovasc Med. 2026 May 20;13:1745004. doi: 10.3389/fcvm.2026.1745004 (PMC13229632; doi:10.3389/fcvm.2026.1745004)
Supplement: Supplementary file 1 [file Table1.docx]

SUPPLEMENTARY DATA

STATISTICAL ANALYSIS.

SPORADIC CARDIAC MYXOMA (SCM) vs CONTROL CASES (CC)

Here, you can find all the data on SCM observed for each case. The TABLE 1 below, reports the tumor location, maximum diameter, and age and sex of each patient. One case has an associated tumor that is rarely observed, a lymphoma. This case (ID 1) was excluded from the biological analysis to avoid false positives for PLAG1 and MYC results due to coexitence with another tumor.

The cases reported here signed the consent, following the Helsinki declaration for clinical study

TABLE 1: general features of SCM

| **ID-number** | **sex** | **age** | **place of tumor** | **Max. dimeter (cm)** | **oddities** |
| --- | --- | --- | --- | --- | --- |
| 1 | F | 45 | mitral valve | 3.5 | linfoma |
| 2 | F | 70 | atrium | 4 |  |
| 3 | M | 71 | atrium | 1.7 |  |
| 4 | F | 54 | atrium | 4 |  |
| 5 | F | 54 | atrium | 4 |  |
| 6 | M | 72 | left atrium | 4.3 |  |
| 7 | F | 82 | atrium | 3 |  |
| 8 | F | 78 | atrium | 1.2 |  |
| 9 | M | 87 | not reported | 4 |  |
| 13 | F | 53 | atrium | 1.8 |  |
| 15 | F | 43 | left atrium | 3 |  |
| 16 | M | 72 | atrium | 3 |  |
| 17 | F | 74 | left atrium | 1.9 |  |
| 18 | F | 76 | atrium | 3.5 |  |
| 19 | F | 58 | left atrium | 2.2 |  |

------------------------------------------------------------------------------------------------------------------------

In the following TABLE 2a-b there are the descriptive analysis of SCM . In table 2a evaluate distribution of SCM by sex: the other table (table 2b) there are the average age, average of tumoral diameter, with standard descriptive statistic before the Youden’s index.

TABLE 2a: descriptive statistical analysis

| **Descriptive Statistical analysis for sex** | **Value observed** |
| --- | --- |
| Number of Female (by sex) | 11 |
| Number of Male (by sex) | 4 |
| Female to Male ratio F/M | 2.75/1 |

| *Table 2b:*  *Descriptive Statistics before the Youden’s correction involving SCM and CC PLAG1* | | | | | |
| --- | --- | --- | --- | --- | --- |
|  |  | age | diameter |  |  |
| Valid |  | 15 | 15 |  |  |
| Missing |  | 0 | 0 |  |  |
| Mode |  | 73.93 | 3.718 |  |  |
| Median |  | 71.00 | 3.000 |  |  |
| Mean |  | 65.93 | 3.007 |  |  |
| Std. Error of Mean |  | 3.525 | 0.262 |  |  |
| Std. Deviation |  | 13.65 | 1.015 |  |  |
| Variance |  | 186.4 | 1.029 |  |  |
| Skewness |  | -0.319 | -0.429 |  |  |
| Std. Error of Skewness |  | 0.580 | 0.580 |  |  |
| Kurtosis |  | -1.098 | -1.236 |  |  |
| Std. Error of Kurtosis |  | 1.121 | 1.121 |  |  |
|  | | | | | |
|  | | | | | |

------------------------------------------------------------------------------------------------------------------------

Below, TABLE 3, there are the status of the PLAG1 rearrangement and the GCG for 3' and 5'of 14 SCM, which has been cleared by futile data. For gene copy gain, the percentages have been rounded up or down to the nearest whole number.

TABLE 3: status of PLAG1 in 14 SCM

| **ID n.** | **GENE** | **n. cell with BA** | **Cell count.** | **% SPLIT** | **% SINGLE RED (5')** | **% SINGLE GREEN (3')** | **oddities** |
| --- | --- | --- | --- | --- | --- | --- | --- |
| 1 | PLAG1 | NV | NV | NV | NV | NV | Removed due to other tumor |
| 2 | PLAG1 | 0 | 200 | 0 | 2 | 4 |  |
| 3 | PLAG1 | 3 | 200 | 1,5 | 2 | 5 |  |
| 4 | PLAG1 | 0 | 40 | 0 | 0 | 2 |  |
| 5 | PLAG1 | 0 | 60 | 0 | 2 | 0 |  |
| 6 | PLAG1 | 1 | 170 | 0,59 | 4 | 1 |  |
| 7 | PLAG1 | 0 | 200 | 0 | 0 | 4 |  |
| 8 | PLAG1 | 1 | 200 | 0,5 | 0 | 2 |  |
| 9 | PLAG1 | 0 | 70 | 0 | 0 | 0 |  |
| 13 | PLAG1 | 3 | 200 | 1,5 | 2 | 3 |  |
| 15 | PLAG1 | 1 | 162 | 0,62 | 0 | 3 |  |
| 16 | PLAG1 | 1 | 200 | 0,5 | 2 | 1 |  |
| 17 | PLAG1 | 1 | 200 | 0,5 | 2 | 2 |  |
| 18 | PLAG1 | 4 | 145 | 2,76 | 1 | 8 |  |
| 19 | PLAG1 | 1 | 200 | 0,5 | 2 | 1 |  |

------------------------------------------------------------------------------------------------------------------------

In the following TABLE 4, we report the descriptive stastistic analisys of 14 SCM and 15 CC, before the ROC curve analysis.

TABLE 4: general descriptive statistical analysis of PLAG1 rearrangement before the cut off correction of SCM and CC.

| *Descriptive Statistics of PLAG1 before the Youden’s correction involving SCM and CC* | | | | | |
| --- | --- | --- | --- | --- | --- |
|  |  |  | Sig. | SCM | CC |
| Valid |  |  |  | 14 | 15 |
| Missing |  |  |  | 1 | 0 |
| Mode |  |  |  | 0.466 | 0.038 |
| Median |  |  |  | 0.500 | 0.400 |
| Mean |  |  |  | 0.641 | 0.319 |
| Std. Error of Mean |  |  |  | 0.211 | 0.083 |
| Std. Deviation |  |  |  | 0.789 | 0.323 |
| Variance |  |  |  | 0.623 | 0.104 |
| Skewness |  |  | p=0.002 | 1.725 | 0.626 |
| Std. Error of Skewness |  |  |  | 0.597 | 0.580 |
| Kurtosis |  |  | p=0.011 | 3.123 | -0.369 |
| Std. Error of Kurtosis |  |  |  | 1.154 | 1.121 |
|  | | | | | |
| *Sig:* Significativity | | | | | |

------------------------------------------------------------------------------------------------------------------------

A ROC CURVE ANALISYS.

We performed a ROC curve analysis (FIGURE 1) to determine the PLAG1 sensitivity or specificity threshold at which cases with a positive tumor and PLAG1 BA are considered "Sick," while cases without a tumor are considered "Healthy." The ROC curve help us to determine the Youden’s index and the optimal cutoff.

The following Table 5, shows the values of the variables used to make an ROC curve with an AUC and a Youden’s index.

 FIGURE 1: ROC curve with an AUC and Youden’s index following the table 5 analysis.


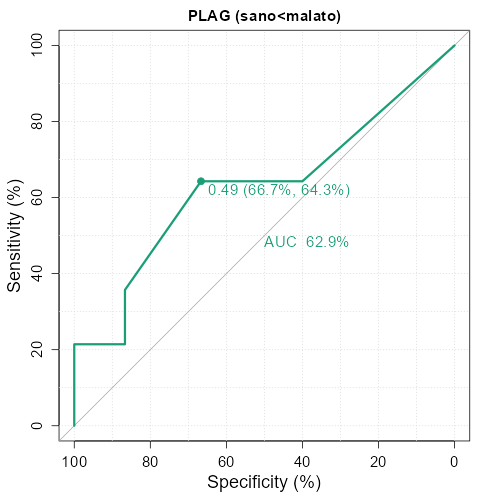


Value of AUC is equal to 62.9 (0.629) and Youden’s index was 0.310 equal to 0,49% (rounded up to 0,5% of cut off) (Sensitivity of 64,3% and Specificity of 66,7% (green dot): Upper bar; PLAG (Healthy<Sick).

| Diagnostic Accuracy - PLAG | | | |
| --- | --- | --- | --- |
|  | | **95% Confidence Interval** | |
|  | **Result** | **Lower** | **Upper** |
| **Sensitivity** | 64.29 % | 35.14 % | 87.24 % |
| **Specificity** | 66.67 % | 38.38 % | 88.18 % |
| **Positive Likelihood Ratio** | 1.929 | 0.853 | 4.36 |
| **Negative Likelihood Ratio** | 0.536 | 0.243 | 1.18 |
| **Prevalence** | 48.28 % | 29.45 % | 67.47 % |
| **Positive Predictive Value** | 64.29 % | 44.34 % | 80.27 % |
| **Negative Predictive Value** | 66.67 % | 47.61 % | 81.48 % |
| **Accuracy** | 65.52 % | 45.67 % | 82.06 % |

| Contingency Table - PLAG-BA | | | | |
| --- | --- | --- | --- | --- |
|  | | **Sick vs Healthy** | |  |
|  |  | **Sick** | **Healthy** | **Total** |
| **PLAG** | **≥0.49** | 9 | 5 | 14 |
|  | **<0.49** | 5 | 10 | 15 |
|  | **Total** | 14 | 15 | 29 |
| *Note.* Based on optimal cut-off (Youden’s Index) | | | | |

| Table 5: Cut-off Coordinates - PLAG-BA | | | |
| --- | --- | --- | --- |
| **Cut-off** | **Sensitivity** | **Specificity** | **Youden's Index** |
| 0.490 | 64.3% | 66.7% | 0.310 |
| 1.250 | 21.4% | 100.0% | 0.214 |
| *Note.* Positive if test ≥ cut-off (direction chosen to maximize AUC). | | | |

———————————————————————————————————————-

ANALYSIS of SCM vs CC after ROC curve and Youden’s index.

After finding a cut-off value using the ROC curve, (TABLE 5) we re-evaluated the data using a Student's t-test and a Welch's t-test for independent samples, as well as an F-test of Snedecor. The latter evaluated a variance difference, while the former two tests evaluated an average difference.

In the the table you can see the p value and in yellow was highlighted a significative value of p.

TABLE 6: Descriptive analysis after Youden’s correction between 14 SCM and the 15 CC.

**Descriptive Statistics**

| *Descriptive Statistics between SCM and CC after Youden’s correction* | | |
| --- | --- | --- |
|  | SCMplag | CCplag. |
| Valid | 14 | 15 |
| Missing | 1 | 0 |
| Mode | 0.006 | -5.633×10^-5^ |
| Median | 0.000 | 0.000 |
| Mean | 0.498 | 0.120 |
| Std. Deviation | 0.849 | 0.319 |
| Variance | 0.722 | 0.102 |
| Skewness | 1.828 | 2.473 |
| Std. Error of Skewness | 0.597 | 0.580 |
| Kurtosis | 2.919 | 4.900 |
| Std. Error of Kurtosis | 1.154 | 1.121 |
|  | | |

| **Descriptive analysis using a cut-off >0.5%** | | | |
| --- | --- | --- | --- |
|  | **SCM** | **CC** |  |
| n. | 14 | 15 |  |
| -Σx | 6.97 | 1.8 |  |
| -Σx^2^ | 12.8501 | 1.640 |  |
| SS(Sum Square) | 9.38 | 1.424 |  |
|  | | | |
| **Student T-test for indipendent sample, one way and a P value** | | | |
| Mean SCM vs mean CC | T | Df | P value |
| 0.3779 | +1.61 | 27 | 0.059 |
| ‍ |  |  |  |
| **Welch T-test for indipendent sample, one way and a P value** | | | |
| Mean SCM vs mean CC | T | Df | P value |
| 0.3779 | 1.56 | 16.38 | 0.068 |
| ‍ |  |  |  |
| **F-test Snedecor (variance analysis) indipendent sample, one tail and a P value** | | | |
| Df1 | Df2 | F-test | P value |
| 13 | 14 | 7.06 | 0.0004 |

------------------------------------------------------------------------------------------------------------------------

In the following table (TABLE 7), there is a value of p before and after the correction using a Bonferroni’s and Benjamini-Hackberg’s test. For a value of α=0.05, the new corrected p-value became p=0.017. Because for the F-test (F=7.06), the observed value of p (0.0004) was lower than 0.017 reported after a correction, the F-test was considered statistically significant.

TABLE 7: correction of p value of F-test.

| **The Correction value for all test used in analysis was:.** | |
| --- | --- |
| **Test Bonferroni for** α=0.05 | **Corrected p value of α=0.017** |
|  |  |
| **Test Benjamini-Hachberg for** α=0.05 | **Corrected p-value of α=0.017** |

Observation:

After applying the Bonferroni and Benjamini-Hockberg corrections, a significant p-value was observed. The variance between SCM and CC is very significant. These data suggest different expressions of SCM and CC, which could be due to different penetrance of the PLAG1 rearrangement.

The high p-value observed in F-test, even after correction, allowed us to avoid a false positive result.

The T-test was not significant or close to it’s limit (p=0.059). These results could be due to a small number of cases evaluated in this study. However, after a correction, a positive trend suggested that PLAG1 break-apart could be an oncogenic mechanism for a SCM origin.

Only 14 cases were found in five years. This small number of cases is due to the disease's rarity. For a correct evaluation of PLAG1 rearrangement, more cases are mandatory in a multicentric study.

Although the number of cases is low, we can say that, a possible link exists between PLAG1 rearrangements and myxomas development.

------------------------------------------------------------------------------------------------------------------------

CONTROL CASES

For a good interpretation of real value of PLAG1 rearrangement, we used the CC that evaluate the PLAG1 rearrangement in normal salivary gland. We choose the salivary gland tissue because is more easier found a rearrangement of PLAG1.

In-fact, the more frequent tumor that show a PLAG1 rearrangement is the pleomorphic adenoma of salivary gland.

In the table below (TABLE 8) there are a rearrangement of PLAG1 in CC. The data are original and reported as observed, without the use of cut-off.

There are a positive cases, the frequency of split (% of BREAK), the number of cells with BA and the minimal cells count for evaluate the BA.

Also the CC were collected after sign the informed consent and following the Helsinky declaration for good clinical study.

TABLE 8: features of PLAG1 in CC

| **case** | **N. count of nuclei** | **n.cell with BA** | **% BREAK** |
| --- | --- | --- | --- |
| 1 | 500 | 2 | 0.4 |
| 2 | 500 | 1 | 0.2 |
| 3 | 500 | 5 | 1 |
| 4 | 500 | 0 | 0 |
| 5 | 500 | 0 | 0 |
| 6 | 200 | 0 | 0 |
| 7 | 200 | 0 | 0 |
| 8 | 200 | 1 | 0.5 |
| 9 | 200 | 0 | 0 |
| 10 | 200 | 0 | 0 |
| 11 | 200 | 1 | 0.5 |
| 12 | 210 | 1 | 0.48 |
| 13 | 200 | 1 | 0.5 |
| 14 | 501 | 2 | 0.4 |
| 15 | 500 | 4 | 0.8 |
